# Genomic insights into the persistence of Nubian giraffe (*Giraffa camelopardalis camelopardalis*) in conflict-affected South Sudan

**DOI:** 10.1038/s41598-026-66559-1

**Published:** 2026-09-05

**Authors:** Sam van Giessen, David Prochotta, Menno Jeroen de Jong, Richard Harvey, Megan Claase, Julian Fennessy, Axel Janke

**Affiliations:** 1 Institute for Biodiversity and Ecosystem Dynamics, University of Amsterdam, P.O. Box 94240, Amsterdam, The Netherlands; 2 Senckenberg Biodiversity and Climate Research Centre (BiK-F), Georg-Voigt-Strasse 14-16, Frankfurt am Main, Germany; 3 Wildscapes Veterinary and Conservation Services, Hoedspruit, South Africa; 4 African Parks Network, Synergy Suites, Juba, South Sudan; 5 Giraffe Conservation Foundation, PO Box 86099, Eros, Windhoek, Namibia; 6 School of Biology and Environmental Science, University College Dublin, Dublin, Ireland; 7 Institute for Ecology, Evolution and Diversity, Goethe University, Max-von-Laue-Strasse. 9, Frankfurt am Main, Germany

**Keywords:** Ecology, Ecology, Evolution, Genetics

## Abstract

Armed conflicts can severely disrupt wildlife conservation and management, yet their long-term genomic consequences remain poorly understood. South Sudan has experienced decades of conflict that have limited conservation efforts and prevented genomic assessment of its fauna, including the endangered subspecies of Nubian giraffe (*Giraffa camelopardalis camelopardalis*). Due to long-standing logistical and political challenges, populations from South Sudan have remained largely unsampled. The Nubian giraffe represents a critical conservation unit and new sampling efforts provide an opportunity to investigate its genomic diversity and potential genetic isolation by the White Nile River, a hypothesized gene flow barrier. Here, we present genomic data from 30 individuals sampled in Boma and Badingilo National Parks in eastern South Sudan. Adding these sequences to existing genomic data reveals genetically distinct groups within the Nubian giraffe according to three regions: Ethiopia-South Sudan, Kenya, Uganda. Despite limited wildlife management due to economic and political constraints in South Sudan, the Nubian giraffe populations have maintained high heterozygosity (He ≈ 0.14%) and minimal evidence of inbreeding (mean F_ROH_ ≈ 0.15) compared to Kenya’s Nubian giraffe populations. Contrary to expectations, our results reveal measurable gene flow across the White Nile between Nubian giraffe and Kordofan giraffe (*G. c. antiquorum*). These findings highlight South Sudanese Nubian giraffe as a population that retained genetic diversity and conservation efforts should be enhanced where feasible to ensure this stronghold long-term.

**Supplementary Information:**

The online version contains supplementary material available at https://doi.org/10.1038/s41598-026-66559-1.

## Introduction

Armed conflict is one of the greatest predictors of wildlife population declines in protected areas in Africa, impacting 71% of African protected areas during the second half of the 20th century^1,2^. One country facing these challenges is South Sudan, notable for having the world’s highest human population growth rate, experiencing prolonged periods of civil unrest in the past century, disrupting the country’s wildlife management and increasing illegal poaching, primarily for bushmeat^3–5^.

Civil unrest, lack of political will, and no formal conservation protection are potentially detrimental to local wildlife populations^1,6^. Some ungulate species seem to have persisted in South Sudan despite conflict e.g. white-eared kob (*Kobus kob leucotis*)^7^ and tiang (*Damaliscus lunatus tiang*)^8^. Nonetheless, during the conflict between 1985 and 2015, Nubian giraffe (*Giraffa camelopardalis camelopardalis*) in Ethiopia and South Sudan suffered a 95% population decline^3^. In 2025, an estimated 235 Nubian giraffe inhabit a relatively extensive range across South Sudan but are restricted to isolated pockets^9^.

The Boma-Badingilo-Jonglei landscape in eastern South Sudan encompasses an open landscape of 200,000 km^2^ consisting of national parks (NPs) and community areas^10,11^. The area is one of the largest conservation areas in Africa and supports the world’s largest terrestrial mammal migration^10,12^. However, historically due to conflict limited active management has existed in South Sudan^4^.

Besides South Sudan, Nubian giraffe currently inhabit Ethiopia, Kenya and Uganda^9^. In Uganda, all six populations persist in protected areas, with all but one having been augmented or (re)introduced, while in Kenya most individuals were introduced from western Kenya^13–16^. The small founder sizes and genetic isolation of these populations raise concerns over potential inbreeding. Conservation translocations have ultimately increased Nubian giraffe numbers and range within Kenya and Uganda, the former by ~ 700%^15^. Despite the recent bottleneck, relatively high genetic diversity has been described for Kenyan and Ugandan populations^17^. With little known about the Nubian giraffe in Ethiopia and South Sudan, largely due to inaccessibility, the northern giraffe population in that landscape may hold valuable genetic diversity. Maintaining this genetic diversity may be an important conservation priority, essential to avoid future genetic erosion and ensure long-term adaptability of the region’s populations^18^.

Giraffe (*Giraffa* spp.) were recognized by the IUCN in August 2025 as four distinct species and seven subspecies with varying degrees of threat amongst them^19^. This announcement was largely based on genomic^17,21–24^ and morphological studies^25,26^. Among the four species, the northern giraffe (*G. camelopardalis*) is critically endangered, with approximately 7,000 individuals^9^ distributed within three subspecies: Kordofan (*G. c. antiquorum*), Nubian (*G. c. camelopardalis*), and West African (*G. c. peralta*). It is important to note that the Rothschild’s giraffe (*G. c. rothschildi*) was subsumed into the Nubian giraffe as part of recent findings^14,21^.

**Fig. 1:**
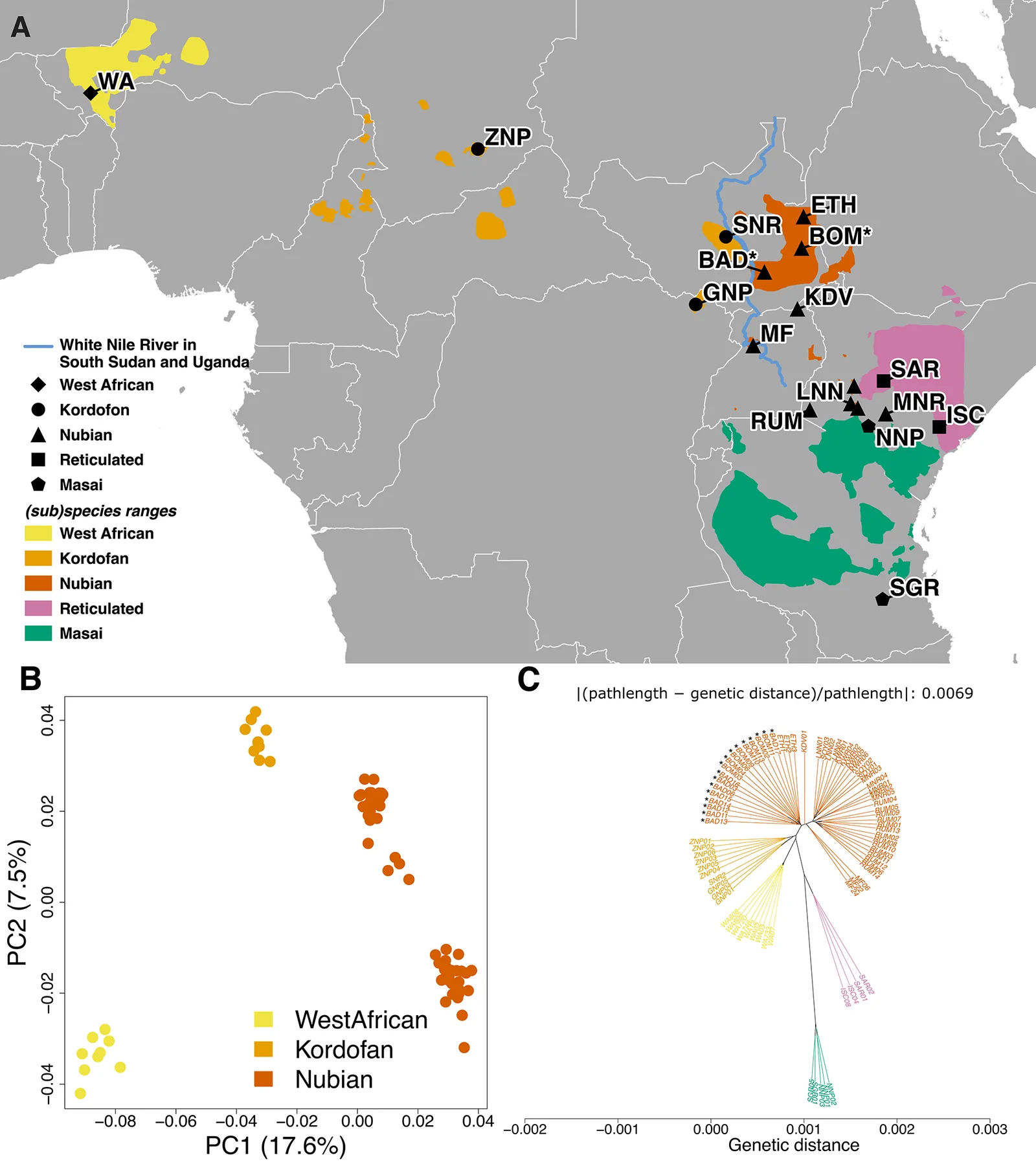
Cross-range sampling and structure analyses show three genetic clusters within Nubian giraffe*.* (**A**) Range map of five giraffe (sub)species with all included sampling locations of this study, for full population names and number of samples per population see Supplementary Table S1 online. The new South Sudan sampling locations in this study, Boma National Park (BOM) and Badingilo National Park (BAD), are marked with an asterisk (*). A section of the White Nile (blue line) splits the range of the Kordofan and Nubian giraffe and is suggested as a possible genetic barrier. Map was created in QGIS v3.40.4^30^ with the current Marneweck et al. (2025) distribution map of Giraffa spp. (**B**) PCoA plot of northern giraffe. The analysis, excluding the outgroup for better resolution, confirms the dendrogram analysis and identifies the same clusters according to subspecies and geographic origin (labels; see Supplementary Table S1 online). (**C**) Distance-based dendrogram of all analysed giraffe individuals in this study. The BioNJ dendrogram is based on genetic dissimilarity between individuals. It shows the separation into the three giraffe species present in the dataset; Masai, reticulated, and northern giraffe, and also shows differentiation between the three subspecies of the northern giraffe. Within the Nubian giraffe three regions of origin are obvious; Ethiopia-South Sudan, Kenya, and Uganda. Colour coding as in A. Origin of individuals: see Supplementary Table S2 online*.*

The known ranges of the Kordofan and Nubian giraffe meet in central South Sudan, with the White Nile River and Sudd swamp acting as potential barriers to gene flow^24^(Fig. 1A). As giraffe reportedly avoid large rivers, it has been suggested that the White Nile River creates a historic genetic divide with limited introgression^24,27,28^. Understanding gene flow between different giraffe taxa is vital for defining taxonomic and geographic conservation units. Evidence of historic Nubian ancestry in reticulated giraffe (*G. reticulata*)^17^ shows historical connectivity between the two species, whilst recent (re)introductions have boosted numerous populations of all four giraffe species across the continent, including across East Africa^9,29^.

More genomic data collection and analysis of the poorly known and inaccessible Nubian giraffe in South Sudan can help better clarify their genetic health, population structure, past bottlenecks, and viability for long-term conservation efforts. This study forms part of the Giraffe Conservation Foundation, GCF/BiK Senckenberg range-wide genome project and aims to continue to inform future conservation management strategies across Africa. This study adds 30 new genomes from Nubian giraffe individuals collected from across the Boma-Badingilo (BOM-BAD) landscape in South Sudan and aims to assess the Nubian giraffe’s: (1) genetic diversity, (2) population structure, and (3) gene flow across the White Nile River of South Sudan.

## Materials and methods

### Sampling, DNA isolation and whole genome sequencing

Skin biopsies from 30 Nubian giraffe were obtained in South Sudan by GCF and partners as part of a GPS satellite tagging operation in collaboration with African Parks and the Government of South Sudan. All sampling was approved by the government, and followed GCF’s IACUC protocol. Importantly, CITES export permit was obtained from South Sudan (No 46/2024) and CITES import permit from Germany (DE-E-04620/23).

All samples were preserved in ≥ 90% ethanol at ambient temperature in the field, and then subsequently stored at + 4 °C. DNA was extracted using the Qiagen DNeasy Blood & Tissue Kit (Qiagen, Hilden, Germany), and then sequenced by BGI for short read, paired end sequencing.

Genome data were also included from other wild *Giraffa*: Nubian (*n* = 40), Kordofan (*n* = 10), West African (*n* = 10), reticulated (*n* = 5), and Masai (*n* = 5) giraffe analysed in Fennessy et al., 2016; Coimbra et al., 2021, 2022, 2023; Bertola et al., 2024. All sampling locations were plotted in *QGIS v3.40.4*^30^ along with the current Marneweck et al. (2025) distribution map of *Giraffa* spp. For detailed information on all sequences used in this study see Supplementary Table S2 online.

### Read processing and mapping

For each sample, FASTQ files from multiple sequencing lanes were concatenated into a single pair of R1 and R2 files. The reads were scanned, trimmed and filtered in a four bp long window approach using *fastp v0.24.1*^31^. The polyG tails and adapter sequences were trimmed off using the –cut-tail option. Only reads with a minimum length of 36 bp, less than 5 unknown bases (Ns), and a quality score of ≥ 15 for more than 40% of its bases remained.

The trimmed reads were mapped against a chromosome level reference Masai giraffe genome^32^ using *bwa mem2*^33^ (for scripts used see: https://github.com/mennodejong1986/WGS_data_analyses). Resulting sequence SAM files were coordinate sorted, converted to binary alignment maps (BAM) format, and merged to sample level using *samtools v1.13*^34^. Duplicate reads in these BAM files were then marked using the MarkDuplicates command from *Picard v2.21.7*^35^ which were later filtered out using samtools. Mapping quality was assessed using *Qualimap2*^36^.

### Genotype calling and individuals filtering

The resulting BAM files of all 100 individuals were used for genotype calling using *bcftools mpileup v1.11*^34^, with a masking depth of 5 and a maximum sample depth of 250. The read depth was calculated using the *bcftools query*^34^ and plotted in *R v4.5.1*^37^ (see Supplementary Fig S1 online). Indels were removed from the VCF file, and the VCF file was filtered for a minimum depth of 100 and a maximum depth of 1,800. Three VCF files were generated for different analyses: (1) variants and monomorphic sites, (2) multi and biallelic SNPs, and (3) biallelic SNPs only.

Due to missing SNP data (> 50%) in some individuals, 16 samples were excluded from downstream analyses after running the *filterdata()* function from the R package *SambaR v1.10*^38^ (see https://github.com/mennodejong1986/SambaR). *SambaR’s calckin()* function was used to identify and exclude three individuals with a King Robust score of > 0.1768, thereby excluding first degree relatives or duplicate individuals^39^. One individual (KWR03) had high levels of missing data and high relatedness scores. Thus, 82 individuals remained for analyses.

### Genetic diversity and inbreeding

Genetic diversity was calculated using a sliding window approach with non-overlapping 20 kb windows on the gVCF file, containing both monomorphic and polymorphic sites, using the Darwindow tool^40^ (for scripts used see https://github.com/mennodejong1986/Darwindow). Estimates were compared with genetic diversity estimates obtained with *bcftools stats v1.11*^34^ for validation. Darwindow was further used to identify Runs of Homozygosity (ROHs), blocks of the genome of individuals that are identical by descent^40,41^. Plots from Darwindow were used to validate different heterozygosity thresholds per population (see Supplementary Fig S3 online). A heterozygosity threshold of 0.05 and maximum missingness of 80% per window were used to identify ROHs, with a minimum ROH length of 200 kb.

### Population structure and admixture

A Principal Coordinates Analysis (PCoA) plot was produced using SambaR’s *findstructure()* function based on an internally constructed genetic distance matrix, using SNPs with a maximum missingness of 2% across retained individuals (snpmiss = 0.02) and a minimum spacing of 50 kb between adjacent SNPs to reduce linkage disequilibrium^38^. An admixture analysis was performed on the same SNP dataset, using the LEA R package^42^, in the *findstructure()* wrapper function^38^. To identify the optimal value for K a cross-entropy analysis was performed 50 times by the *LEAace()* function. The admixture analysis was repeated using a thinned dataset where Nubian giraffe were randomly down sampled to 10 individuals.

A BioNJ dendrogram was constructed based on genome-wide pairwise sequence dissimilarity estimates calculated using DIST from the VCF file containing both polymorphic and monomorphic sites^43^. The resulting distance matrix was used as input for SambaR’s *runDIST()* function^38^.

### Gene flow

To detect gene flow, particularly between the Kordofan and Nubian giraffe populations across the White Nile River, a F-branch (F_b_) statistical test was performed using *Dsuite v0.5*^44^. The program used the biallelic SNP dataset together with a distance-based phylogenetic tree, which was created in Sambar’s *findstructure()* function, using the Masai giraffe as an outgroup^38^.

## Results

### DNA isolation and sequencing

Of the 30 new South Sudan samples, DNA of sufficient quality and quantity was isolated for sequencing. The data was complemented with 70 previously published short read sequences (see Supplementary Table S2 online). After filtering and mapping of the reads and subsequent genotype calling, 18 individuals were excluded from further analyses: 16 due to high missing SNP data (> 50%) and three due to high levels of relatedness (king robust score > 0.1768), of whome one individual met both exclusion criteria (KWR03). The remaining 82 genome sequences had an average read depth per site of 5.9–27.8 (x̄ = 12.6) after quality filtering and genotype calling. Specifically, BAD individuals had a mean depth of 21.2× (range: 7.4–27.8×), while BOM individuals had a mean depth of 14.0× (range: 6.7–27.6×) (see Supplementary Fig S2 online). However, individuals with lower sequencing coverage did not show any systematic deviation in the downstream analyses.

### Genetic diversity and inbreeding

**Fig. 2:**
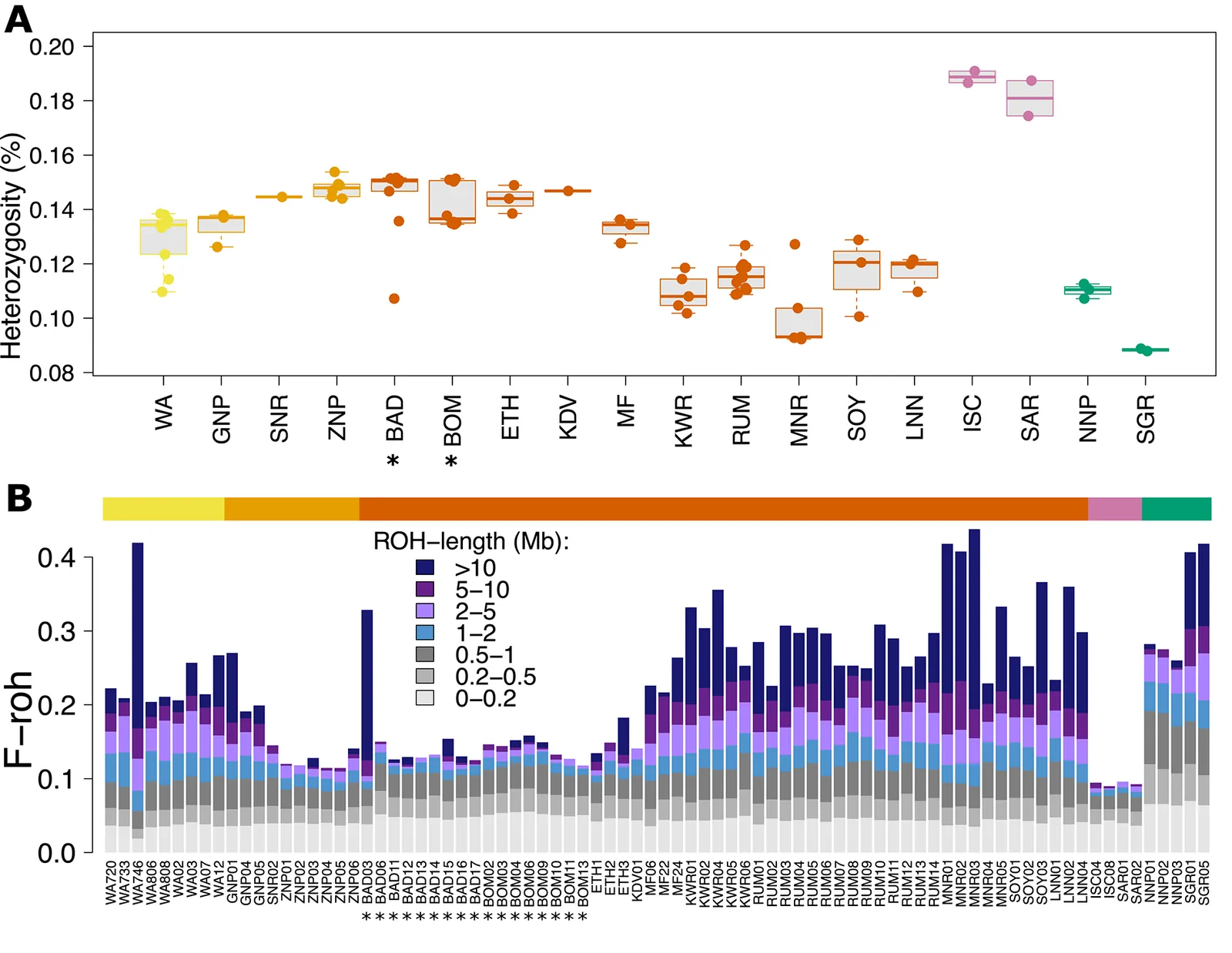
Window-based analyses show high diversity and low levels of inbreeding in Nubian giraffe in Ethiopia (ETH) and South Sudan (BAD, BOM).** (A**) Boxplot of the genome wide heterozygosity levels of all populations. Populations correspond to the locations in Fig. 1A, for full population names and number of samples per population see Supplementary Table S1 online. The groups are colored according to (sub)species assignment (yellow= *G.c. peralta*, light orange = *G.c. antiquorum*, dark orange = *G.c. camelopardalis*, pink = *G. reticulata*, green = *G. tippelskirchi*). (**B**) Barplot of the inbreeding coefficients (F_ROH_) calculated by Darwindow. Each bar represents an individual identified by sample name as in Supplementary Table S2 and the different coloured segments of the bars represent the contribution of different ROH length classes to the overall F_ROH_ of the individuals. The coloured horizontal bar (colour coding as in Fig. 1) shows the (sub) species designations.

With one exception, individual BAD03 (He = 0.11%), the genome-wide heterozygosity (He) of the newly genome sequenced individuals from South Sudan (BAD&BOM) (He = 0.14%) was within the range of other Nubian giraffe populations and slightly higher than the average northern giraffe (He = 0.12%) (Fig. 2A). By comparison, the reticulated giraffe has the highest reported He (0.18%) and the Masai giraffe the lowest He (0.10%).

ROHs were generally shorter in almost all South Sudanese individuals (x̄ F_ROH_ = 0.15) compared to other northern giraffe (x̄ F_ROH_ = 0.28), except for BAD03 (F_ROH_ = 0.33) (Fig. 2B). Most individuals had few long ROHs that are > 5 Mb and ROHs > 10 Mb were rare, except for individual BAD03, which had many very long ROHs.

### Population structure and admixture

A BioNJ dendrogram based on pairwise nucleotide differences of all individuals (Fig. 1C), supports a clear separation into three giraffe species (Masai, northern and reticulated), and within the northern giraffe individuals, distinct clustering into the three subspecies (Kordofan, Nubian and West African). Nubian giraffe further subdivide according to the three regions of origin: Ethiopia-South Sudan, Kenya, and Uganda. Population splits are assumed to be relatively recent. Therefore, inner branch lengths primarily reflect levels of genetic drift rather than the accumulation of substitutions. Longer internal branches typically indicate increased genetic drift, reflecting reduced genetic diversity within a group. In particular, the West African giraffe form a clade characterized by a notably long internal branch, consistent with the genetic signature of a population bottleneck. Similarly, extended internal branches are observed within the Nubian giraffe populations of Kenya and Uganda, which likely also reflect recent bottleneck events. Additionally, pairs of closely related internal branches likely represent individuals with close kinship.

A PCoA plot for the northern giraffe individuals shows the grouping of the three northern giraffe subspecies along the PC1 and PC2 axes (Fig. 1B, see Supplementary Fig S4 online). Within the Nubian giraffe, the three clusters correspond to the geographic origin of the individuals: one cluster for Ethiopia-South Sudan, Kenya, and Uganda. The individuals from Ethiopia and South Sudan are not distinguishable according to their origin in both the dendrogram and PCoA, and as such form a homogenous population (Fig. 1B,C).

**Fig. 3:**
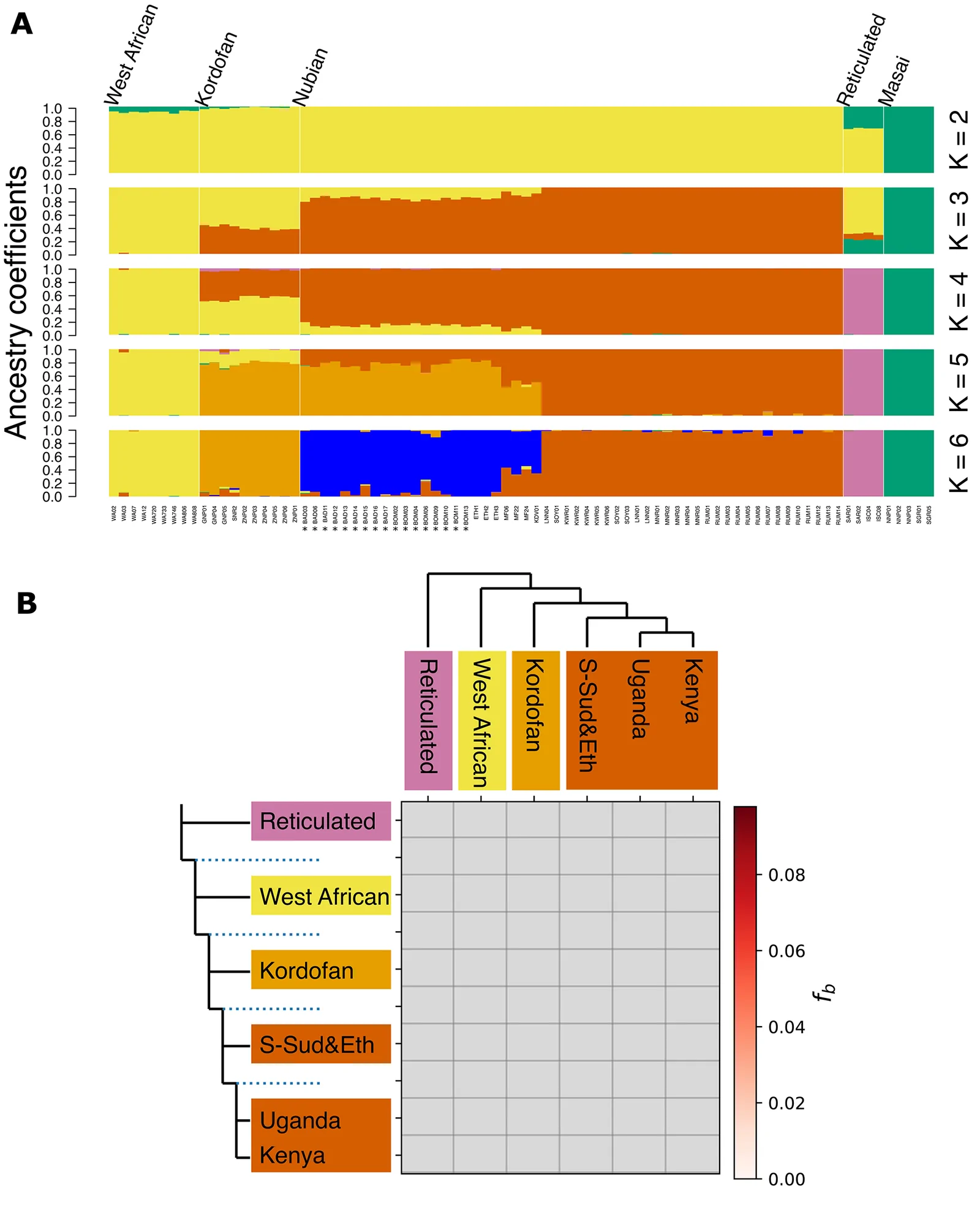
Admixture and geneflow indicate past connectivity between Nubian and Kordofan populations and between Nubian and West African populations.(**A**) Admixture plot for K = 2-6. Each vertical bar represents one individual from the five (Sub)species; Kordofan, Masai, Nubian, reticulated, and West African giraffe. The colours represent the division of the individuals into a specified number of genetic clusters (K). Individuals with mixed coloured bars indicate admixture, or artifacts of admixture when too few genetic clusters are imposed on the data. At the optimal K = 5 the Kordofan, Masai, reticulated, and West African giraffe show little admixture. Within the Nubian giraffe the individuals from Ethiopia, South Sudan and Uganda show admixed ancestry with the Kordofan giraffe. (**B**) Heatmap of estimated gene flow from Dsuite tests between major clades found in the dataset (Fig. 1B,C), using the Masai giraffe as an outgroup. The clades seen in the heatmap are Reticulated (ISC, SAR) (N = 4), West African (WA) (N = 9), Kordofan (ZNP, GNP, SNR) (N = 10), Nubian in South Sudan and Ethiopia (BAD, BOM, ETH) (N = 20), Nubian in Uganda (MF, KDV) (N = 4), and Nubian in Kenya (RUM, MNR, SOY, KWR, LNN) (N = 30). The darker coloured cells indicate higher levels of gene flow (f_b_) between populations, the colours are assigned according to subspecies as in Fig 1A.

The admixture analysis groups the individuals according to the number of given genomic clusters, the K-value (2–6). At K = 2 and K = 3 the individuals grouped according to their respective species (Fig. 3A). The indicated admixture from Masai at K = 2 into reticulated giraffe, and from West African into reticulated giraffe at K = 3, appears to be an artifact as almost all signals of admixture among the species disappear at K > 3.

At K = 5, the optimal value of K (see Supplementary Fig. 6), the Masai and reticulated giraffe form distinct and almost homogeneous clusters with very little admixture signal (Fig. 3A). Nubian giraffe from Ethiopia, South Sudan and Uganda show almost completely admixed ancestry between the Kenyan Nubian giraffe and the Kordofan giraffe. When adding another cluster to the model for K = 6, the Ethiopian and South Sudanese Nubian giraffe form their own genetic cluster, and the Ugandan Nubian giraffe appear to be of admixed ancestry of the Ethiopian-South Sudanese group and the Kenyan Nubian giraffe. The same pattern of shared ancestry between Nubian and Kordofan giraffe was observed in the down sampled dataset at K = 4 and K = 5 (See Supplementary Figs. 7,8).

### Gene flow

While using the genetic clusters identified in this study (Fig. 1B,C) as branches, the D-suite analysis revealed historical gene flow between the Kordofan and West African giraffe (Fig. 3B). Gene flow was also found between the Nubian giraffe from South Sudan and Ethiopia to both the Kordofan and West African groups, showing that populations on opposite sides of the White Nile River exhibited evidence of historical gene flow. Using all populations as tips in the distance-based phylogenetic tree, allowed for a more fine-scale assessment of gene flow (see Supplementary Fig S9 online). Within the Nubian giraffe in Kenya there was gene flow between the Lake Nakuru NP population (LNN) with those from South Sudan’s Badingilo NP (BAD) and between two Kenyan populations (RUM & SOY). The Badingilo (BAD) population of South Sudan also showed gene flow with two Nubian populations in Kenya (LNN, SOY) and two in Uganda (KDV, MF). The ancestral branch of the Kordofan populations (ZNP, GNP, SNR) showed gene flow with the West African giraffe populations from Niger (WA).

## Discussion

This study presents the first detailed genomic assessment of Nubian giraffe living in South Sudan. *Giraffa* spp. conservation has greatly benefitted from a marked increase in targeted science over the past two decades^17,21–24,45^, including large-scale tissue sampling from across all major populations in Africa. By incorporating 30 new samples from the previously under sampled conflict-affected South Sudan, this study fills a critical sampling gap.

Our study reveals clear population structure supporting the subdivision of the northern giraffe into three subspecies with clear genetic clustering in the Nubian giraffe across the three regions: Ethiopia-South Sudan, Kenya, and Uganda (Fig. 1B,C). These three regions’ Nubian giraffe populations could be considered separate Evolutionarily Significant Units (ESUs) in future conservation planning. Nubian giraffe from Ethiopia-South Sudan showed high levels of historical Kordofan ancestry (Fig. 3B), possibly resulting in their genetic distinctness within Nubian giraffe. The clustering of Ethiopia-South Sudan giraffe supports the ecological observations of unrestricted transboundary movement within the Boma-Badingilo-Gambella landscape^3^, although this has not been observed in recent years. Historically, the Nubian giraffe now living in South Sudan and Ethiopia were classified as *G.c. rothschildi*^14,49^, before being subsumed under the Nubian giraffe^19,21^. Despite being genetically distinguishable from other Nubian giraffe (Fig. 1B,C), the presented data of South Sudan’s Nubian giraffe does not provide sufficient evidence to warrant their re-evaluation.

The Boma and Badingilo NP samples show high levels of heterozygosity (Fig. 2A), similar to levels of Nubian giraffe from Gambella NP (ETH), Kidepo Valley NP (KDV) and Kordofan giraffe populations (SNR and ZNP). In contrast, other Nubian giraffe populations in Kenya (KWR, RUM, MNR, SOY, LNN) display substantially lower genetic diversity – possibly a result of them largely being translocated to their respective current populations from the one source population in western Kenya^15^. High genetic diversity was unexpected in South Sudan since limited wildlife management and protection existed due to civil unrest and the Nubian giraffe has experienced a population decline of approximately 95% from 1985 to 2015^3,4^. Reticulated giraffe retain the highest levels of genetic diversity and Masai giraffe show the lowest levels of genetic diversity, supporting earlier studies^23,24^. However, none of the results indicate a direct threat to the viability of the Nubian giraffe populations due to low genetic diversity. Instead, the relatively high levels of genetic diversity could indicate that those in South Sudan could be a valuable gene pool for possible future reintroductions of Nubian giraffe, in areas where Nubian giraffe are locally extinct. Alternatively, the consequences of genetic drift following the severe bottleneck may not be yet observable^46^.

The high levels of genetic diversity in South Sudan’s Nubian giraffe correspond to small signs of realized inbreeding (x̄ F_ROH_= 0.15) for the majority, similar to the connected Gambella NP (ETH) population (x̄ F_ROH_ = 0.16) (Fig. 2B). Because recombination breaks down ROHs over generations, long ROHs indicate recent inbreeding and small ROHs signal more ancient inbreeding^41^. The difference in overall F_ROH_ between the Ethiopia-South Sudan populations and the Nubian populations from Kenya is almost entirely made up of very long ROHs (> 10 Mb) (Fig. 2B), thus indicating that recent inbreeding events have most likely caused the higher levels of realized inbreeding and subsequent reduced genetic diversity. Accordingly, background heterozygosity levels outside of ROHs are very similar between all Nubian giraffe populations but recent inbreeding has likely decreased genetic diversity in the Nubian giraffe in Kenya (see Supplementary Fig S5 online).

This relatively low realized inbreeding in South Sudan’s Nubian giraffe was unexpected since limited conservation management has occurred there due to large scale conflicts in the past decades^3,4^. In contrast, large scale conflicts are reported to have greatly impacted genetic diversity and increased inbreeding depression in other large mammals^47^. The large open areas of Ethiopia and South Sudan where Nubian giraffe inhabit provide an environment where they are free to roam^3^. This in contrast to many of the populations in Kenya who predominantly inhabit small, fenced areas, with limited effective population sizes and derived from limited founder stock through conservation translocations, thus increasing genetic drift^14,15,20,48,49^. The genomic resilience of the Nubian giraffe highlights their conservation importance in the vast, unfenced Boma–Badingilo–Gambella landscape.

The Nubian giraffe is geographically isolated from the Kordofan giraffe by the White Nile River (Fig. 1A). Both mitochondrial^28^ and nuclear^24^ genetic data support this theory, noting that the White Nile River has acted as a possible genetic barrier. Our new findings from a previously unsampled border region in South Sudan reveal signals of gene flow between Kordofan (ZNP, GNP, SNR) and West African (WA) populations and Nubian giraffe populations in Ethiopia (ETH) and South Sudan (BOM, BAD) (Fig. 3B), although not observed in the smaller population level analysis (see Supplementary Fig S9 online). Finding these signs of historic gene flow was unexpected between these groups living on opposite sides of the White Nile River. Physical barriers such as large or quickly flowing water bodies^27,50^ are not the only expected limitation to natural gene flow between giraffe populations, reproductive barriers have also been suggested by assortative mating based on different pelage patterns and differences in temporal mating preferences based on local rain patterns^51,52^.

## Conclusion

South Sudan’s Nubian giraffe represent a genomic stronghold for the subspecies, exhibiting high levels of genetic diversity and relatively low inbreeding compared to other wild *Giraffa* populations. Given the relatively small population size, large-scale population decline, and limited conservation management, these results were unexpected. Importantly for conservation, their unique genetic status should be conserved in South Sudan long-term. While the White Nile River does not form an absolute genetic boundary between the Kordofan and Nubian populations, the detected gene flow highlights historical movements of individual giraffe across it. Importantly, our findings highlight that Nubian giraffe are genetically distinct amongst three regions: Ethiopia-South Sudan, Kenya, and Uganda. The expansion of wildlife genomics to sequence all major populations of *Giraffa* in the future provides a powerful tool for assessing true biodiversity and defining ESUs within (sub)species, providing critical guidance for the planning and coordination of future conservation management.

## Supplementary Information

Below is the link to the electronic supplementary material.

Supplementary Material 1

Supplementary Material 2

Supplementary Material 3

Supplementary Material 4

Supplementary Material 5

Supplementary Material 6

Supplementary Material 7

Supplementary Material 8

## Data Availability

Sequence data is available at NCBI Short Read Archive through BioProject PRJNA1419911. The VCF file containing all variant sites is available in the Zenodo repository, https://doi.org/10.5281/zenodo.21776240 .

## References

[1] Daskin, J. H. & Pringle, R. M. Warfare and wildlife declines in Africa’s protected areas. *Nature* **553**, 328–332 (2018). 29320475 10.1038/nature25194

[2] Stalmans, M. E., Massad, T. J., Peel, M. J. S., Tarnita, C. E. & Pringle, R. M. War-induced collapse and asymmetric recovery of large-mammal populations in Gorongosa National Park, Mozambique. *PLoS ONE* **14**, e0212864 (2019). 30865663 10.1371/journal.pone.0212864 PMC6415879

[3] Wube, T., Doherty, J. B., Fennessy, J. & Marais, A. Giraffa camelopardalis ssp. camelopardalis. *IUCN Red List. Threatened Species*. **2018**, eT88420707A88420710. 10.2305/IUCN.UK.2018-2.RLTS.T88420707A88420710.en (2018).

[4] Day, C. & Garside, A. Wildlife Management in South Sudan, 1901–2021: Conservation amidst Conflict. *Afr. Stud. Rev.* **67**, 610–631 (2024).

[5] Central Intelligence Agency. South Sudan. *The World Factbook 2024* https://www.cia.gov/the-world-factbook/field/population-growth-rate/country-comparison/ (2024).

[6] Gaynor, K. M. et al. War and wildlife: Linking armed conflict to conservation. *Front. Ecol. Environ.* **14**, 533–542 (2016).

[7] IUCN SSC Antelope Specialist Group. Kobus kob ssp. leucotis. *IUCN Red List. Threatened Species*. **2016**, eT11042A50190165. 10.2305/IUCN.UK.2016-2.RLTS.T11042A50190165.en (2016).

[8] IUCN SSC Antelope Specialist Group. Damaliscus lunatus ssp. tiang. *IUCN Red List. Threatened Species*. **2017**, eT6242A50185852. 10.2305/IUCN.UK.2017-2.RLTS.T6242A50185852.en (2017).

[9] Marneweck, C. J. et al. State of Giraffe 2025: An update from the Giraffe Africa Database (GAD). 10.5281/zenodo.15688798 (2025).

[10] Morjan, M. D. et al. Armed conflict and development in South Sudan threatens some of Africa’s longest and largest ungulate migrations. *Biodivers. Conserv.* **27**, 365–380 (2018).

[11] IUCN ESARO. The state of protected and conserved areas in Eastern and Southern Africa, 2nd ed. *State of Protected and Conserved Areas Report Series No. 1* 10.2305/VUQC1935 (2024).

[12] Harris, G., Thirgood, S., Hopcraft, J. G. C., Cromsigt, J. P. G. M. & Berger, J. Global decline in aggregated migrations of large terrestrial mammals. *Endanger. Species Res.* **7**, 55–76 (2009).

[13] Brown, M. B., Bolger, D. T. & Fennessy, J. All the eggs in one basket: A countrywide assessment of current and historical giraffe population distribution in Uganda. *Glob. Ecol. Conserv.* **19**, e00612 (2019).

[14] Muller, Z. Rothschild’s giraffe Giraffa *camelopardalis rothschildi* (Linnaeus, 1758) in East Africa: A review of population trends, taxonomy and conservation status. *Afr. J. Ecol.* **57**, 20–30 (2019).

[15] Muneza, A. B. et al. Updated review of the conservation status of Nubian giraffe (*Giraffa camelopardalis camelopardalis*) in Kenya. *Biodivers. Conserv.* **33**, 1269–1284 (2024).

[16] Brown, M. B. et al. Assessing the success of conservation translocation establishment: Post-translocation demography of Nubian giraffe (*Giraffa camelopardalis camelopardalis*) in Uganda. *Conserv. Sci. Pract.* 10.1111/csp2.70127 (2025).

[17] Coimbra, R. T. F. et al. Genomic analysis reveals limited hybridization among three giraffe species in Kenya. *BMC Biol.* **21, **215. (2023). 37833744 10.1186/s12915-023-01722-y PMC10576358

[18] Frankham, R. Genetics and extinction. *Biol. Conserv.* **126**, 131–140 (2005).

[19] IUCN SSC Giraffe and Okapi Specialist Group Taxonomic Task Force. An evaluation of the taxonomic status of giraffe (*Giraffa spp.*). https://iucn.org/sites/default/files/2025-08/gosgtaxonomictask-force_iucn_giraffetaxonomyassessment_final.pdf (2025).

[20] Muller, Z. Population structure of giraffes is affected by management in the Great Rift Valley, Kenya. *PLoS One*. **13**, e0189678 (2018). 29298338 10.1371/journal.pone.0189678 PMC5751992

[21] Fennessy, J. et al. Multi-locus Analyses Reveal Four Giraffe Species Instead of One. *Curr. Biol.* **26**, 2543–2549 (2016). 27618261 10.1016/j.cub.2016.07.036

[22] Winter, S., Fennessy, J. & Janke, A. Limited introgression supports division of giraffe into four species. *Ecol. Evol.* **8**, 10156–10165 (2018). 30397455 10.1002/ece3.4490 PMC6206193

[23] Coimbra, R. T. F. et al. Whole-genome analysis of giraffe supports four distinct species. *Curr. Biol.* **31**, 2929–2938e5 (2021). 33957077 10.1016/j.cub.2021.04.033

[24] Bertola, L. D. et al. Giraffe lineages are shaped by major ancient admixture events. *Curr. Biol.* **34**, 1576-1586e.e5 (2024). 38479386 10.1016/j.cub.2024.02.051

[25] Kargopoulos, N. et al. Heads up–four *Giraffa* species have distinct cranial morphology. *PLoS One* **19**, e0315043 (2024). 39700177 10.1371/journal.pone.0315043 PMC11658530

[26] Muneza, A. B. et al. Effective conservation and management of giraffe require adopting recent advances of their taxonomy. *Biodivers. Conserv.* **34**, 1211–1229 (2025).

[27] Henderson, D. M. & Naish, D. Predicting the buoyancy, equilibrium and potential swimming ability of giraffes by computational analysis. *J. Theor. Biol.* **265**, 151–159 (2010). 20385144 10.1016/j.jtbi.2010.04.007

[28] Petzold, A. et al. First insights into past biodiversity of giraffes based on mitochondrial sequences from museum specimens. *Eur. J. Taxonomy*. 10.5852/ejt.2020.703 (2020).

[29] Gašparová, K. et al. Saving the Last West African Giraffe Population: A Review of Its Conservation Status and Management. *Animals* **14**, 702 (2024). 38473087 10.3390/ani14050702 PMC10930505

[30] Dawson, N. et al. qgis/QGIS: 3.40.4. *Zenodo*10.5281/zenodo.14906438 (2025).

[31] Chen, S., Zhou, Y., Chen, Y. & Gu, J. fastp: an ultra-fast all-in-one FASTQ preprocessor. *Bioinformatics* **34**, i884–i890 (2018). 30423086 10.1093/bioinformatics/bty560 PMC6129281

[32] Farré, M. et al. An integrated chromosome-scale genome assembly of the Masai giraffe (*Giraffa camelopardalis tippelskirchi*). *Gigascience* **8**, giz090 (2019). 31367745 10.1093/gigascience/giz090 PMC6669057

[33] Li, H. & Durbin, R. Fast and accurate short read alignment with Burrows–Wheeler transform. *Bioinformatics* **25**, 1754–1760 (2009). 19451168 10.1093/bioinformatics/btp324 PMC2705234

[34] Danecek, P. et al. Twelve years of SAMtools and BCFtools. *GigaScience* **10**, giab008 (2021). 33590861 10.1093/gigascience/giab008 PMC7931819

[35] Picard toolkit. *Broad Institute, GitHub repository* https://broadinstitute.github.io/picard/ (2019).

[36] Okonechnikov, K., Conesa, A. & García-Alcalde, F. Qualimap 2: advanced multi-sample quality control for high-throughput sequencing data. *Bioinformatics* **32**, 292–294 (2016). 26428292 10.1093/bioinformatics/btv566 PMC4708105

[37] R Core Team. *R: A Language and Environment for Statistical Computing* https://www.R-project.org/ (R Foundation for Statistical Computing, 2025).

[38] de Jong, M. J., de Jong, J. F., Hoelzel, A. R. & Janke, A. SambaR: An R package for fast, easy and reproducible population-genetic analyses of biallelic SNP data sets. *Mol. Ecol. Resour.* **21**, 1369–1379 (2021). 33503314 10.1111/1755-0998.13339

[39] Manichaikul, A. et al. Robust relationship inference in genome-wide association studies. *Bioinformatics* **26**, 2867–2873 (2010). 20926424 10.1093/bioinformatics/btq559 PMC3025716

[40] de Jong, M. J. et al. Range-wide whole-genome resequencing of the brown bear reveals drivers of intraspecies divergence. *Commun. Biol.* **6**, 1–16 (2023). 36746982 10.1038/s42003-023-04514-w PMC9902616

[41] Ceballos, F. C., Joshi, P. K., Clark, D. W., Ramsay, M. & Wilson, J. F. Runs of homozygosity: Windows into population history and trait architecture. *Nat. Rev. Genet.* **19**, 220–234 (2018). 29335644 10.1038/nrg.2017.109

[42] Frichot, E. & François, O. LEA: An R package for landscape and ecological association studies. *Methods Ecol. Evol.* **6**, 925–929 (2015).

[43] de Jong, M. J. & Janke, A. DIST: Distance-based Inference of Species Trees. BioRxiv 10.1101/2025.05.02.651899 (2025).

[44] Malinsky, M., Matschiner, M. & Svardal, H. Dsuite - Fast D-statistics and related admixture evidence from VCF files. *Mol. Ecol. Resour.* **21**, 584–595 (2021). 33012121 10.1111/1755-0998.13265 PMC7116594

[45] Coimbra, R. T. F., Winter, S., Mitchell, B., Fennessy, J. & Janke, A. Conservation Genomics of Two Threatened Subspecies of Northern Giraffe: The West African and the Kordofan Giraffe. *Genes* **13**, 221 (2022). 35205265 10.3390/genes13020221 PMC8872558

[46] Allendorf, F. W., Funk, W. C., Aitken, S. N., Byrne, M. & Luikart, G. Conservation and the Genomics of Populations (3rd ed.). 10.1093/oso/9780198856566.003.0001 (2022). PMC9753819 36540641

[47] Colangelo, P. et al. Genome-wide diversity, population structure and signatures of inbreeding in the African buffalo in Mozambique. *BMC Ecol. Evo*. **24**, 29 (2024). 10.1186/s12862-024-02209-2 PMC10910738 38433185

[48] Brenneman, R. A., Bagine, R. K., Brown, D. M., Ndetei, R. & Louis, E. E. Jr. Implications of closed ecosystem conservation management: The decline of Rothschild’s giraffe (*Giraffa camelopardalis rothschildi*) in Lake Nakuru National Park, Kenya. *Afr. J. Ecol.* **47**, 711–719 (2009).

[49] Fennessy, S., Fennessy, J., Muller, Z., Brown, M. & Marais, A. Giraffa camelopardalis ssp. rothschildi. *IUCN Red List Threatened Species* **2018**, eT174469A51140829. 10.2305/IUCN.UK.2018-2.RLTS.T174469A51140829.en (2018).

[50] Bercovitch, F. B. & Berry, P. S. M. Ecological determinants of herd size in the Thornicroft’s giraffe of Zambia. *Afr. J. Ecol.* **48**, 962–971 (2010).

[51] Brown, D. M. et al. Extensive population genetic structure in the giraffe. *BMC Biol.* **5**, 57 (2007). 18154651 10.1186/1741-7007-5-57 PMC2254591

[52] Thomassen, H. A., Freedman, A. H., Brown, D. M., Buermann, W. & Jacobs, D. K. Regional differences in seasonal timing of rainfall discriminate between genetically distinct East African giraffe taxa. *PLoS One* **8**, e77191 (2013). 24194870 10.1371/journal.pone.0077191 PMC3806738

