# Supplementary material for: Genomic insights into the persistence of Nubian giraffe (Giraffa camelopardalis camelopardalis) in conflict-affected South Sudan
Source: Sci Rep. 2026 Sep 5;16:27825. doi: 10.1038/s41598-026-66559-1 (PMC13546283; doi:10.1038/s41598-026-66559-1)
Supplement: Supplementary file 1 [file 41598_2026_66559_MOESM2_ESM.pdf]

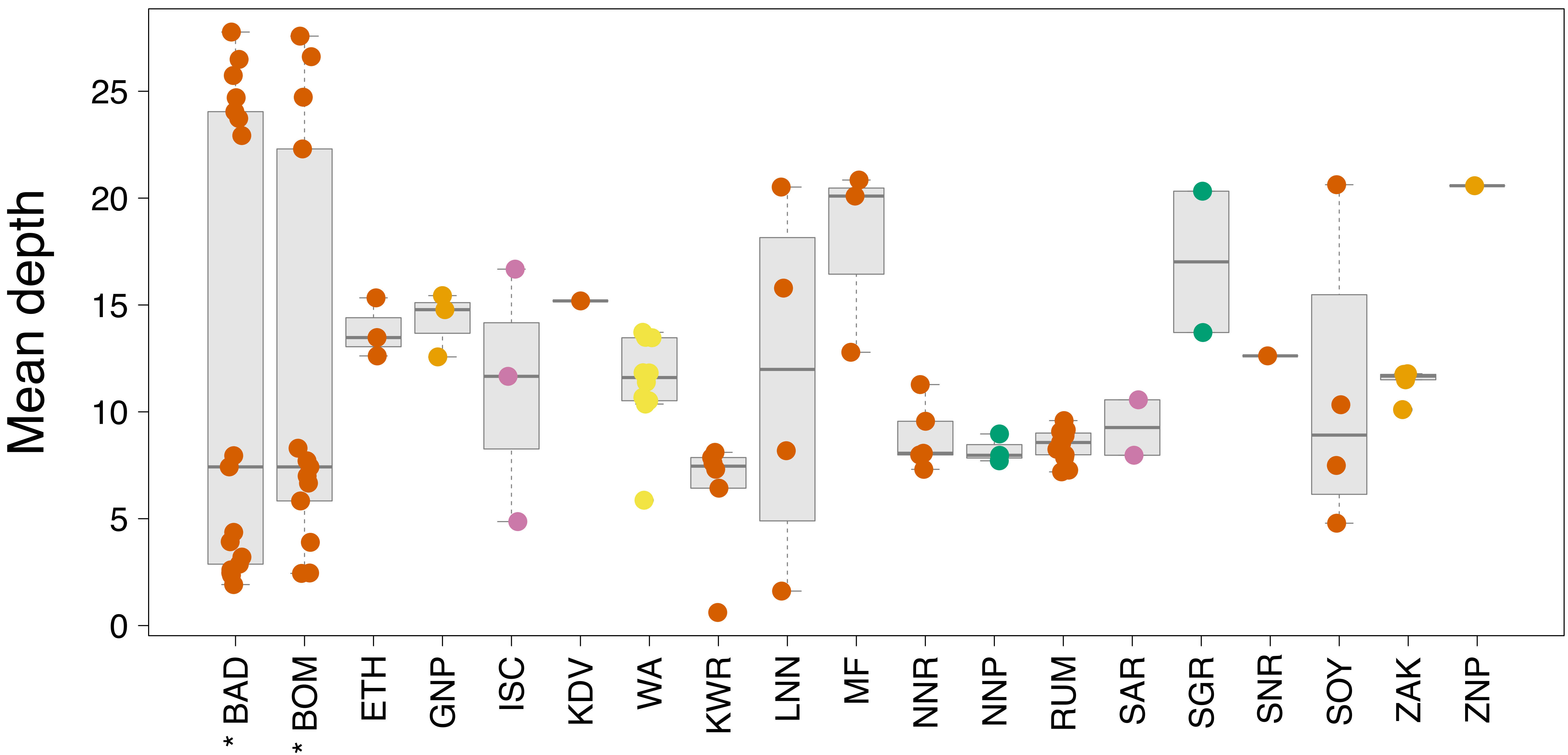

Mean depth
0
5
10
15
20
25
* BAD
* BOM
ETH
GNP
ISC
KDV
WA
KWR
LNN
MF
NNR
NNP
RUM
SAR
SGR
SNR
SOY
ZAK
ZNP
