# Supplementary material for: Genomic insights into the persistence of Nubian giraffe (Giraffa camelopardalis camelopardalis) in conflict-affected South Sudan
Source: Sci Rep. 2026 Sep 5;16:27825. doi: 10.1038/s41598-026-66559-1 (PMC13546283; doi:10.1038/s41598-026-66559-1)
Supplement: Supplementary file 2 [file 41598_2026_66559_MOESM3_ESM.pdf]

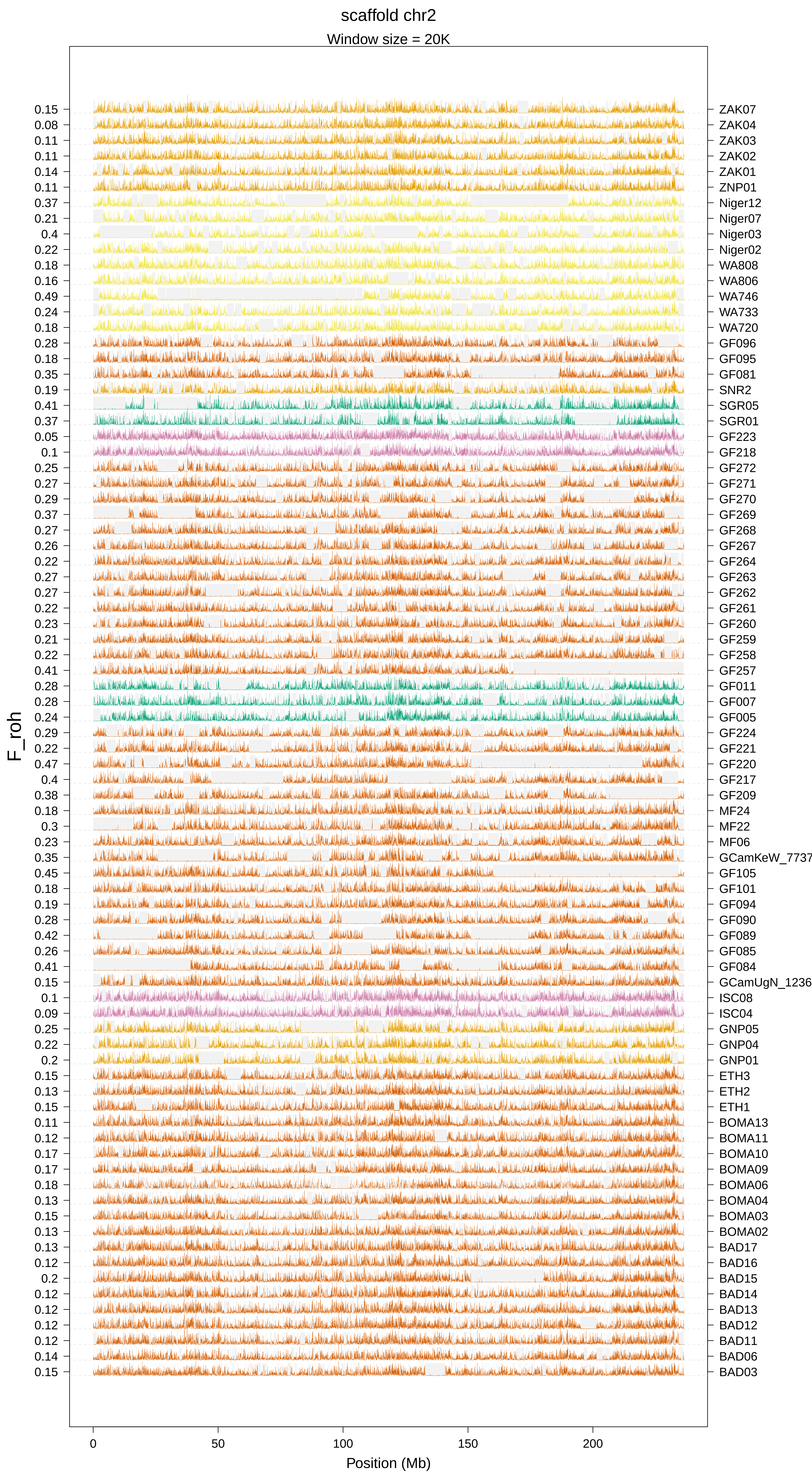
scaffold chr2
Window size = 20K
F_roh
Position (Mb)
0
50
100
150
200
ZAK07 0.15
ZAK04 0.08
ZAK03 0.11
ZAK02 0.11
ZAK01 0.14
ZNP01 0.11
Niger12 0.37
Niger07 0.21
Niger03 0.4
Niger02 0.22
WA808 0.18
WA806 0.16
WA746 0.49
WA733 0.24
WA720 0.18
GF096 0.28
GF095 0.18
GF081 0.35
SNR2 0.19
SGR05 0.41
SGR01 0.37
GF223 0.05
GF218 0.1
GF272 0.25
GF271 0.27
GF270 0.29
GF269 0.37
GF268 0.27
GF267 0.26
GF264 0.22
GF263 0.27
GF262 0.27
GF261 0.22
GF260 0.23
GF259 0.21
GF258 0.22
GF257 0.41
GF011 0.28
GF007 0.28
GF005 0.24
GF224 0.29
GF221 0.22
GF220 0.47
GF217 0.4
GF209 0.38
MF24 0.18
MF22 0.3
MF06 0.23
GCamKeW_7737 0.35
GF105 0.45
GF101 0.18
GF094 0.19
GF090 0.28
GF089 0.42
GF085 0.26
GF084 0.41
GCamUgN_1236 0.15
ISC08 0.1
ISC04 0.09
GNP05 0.25
GNP04 0.22
GNP01 0.2
ETH3 0.15
ETH2 0.13
ETH1 0.15
BOMA13 0.11
BOMA11 0.12
BOMA10 0.17
BOMA09 0.17
BOMA06 0.18
BOMA04 0.13
BOMA03 0.15
BOMA02 0.13
BAD17 0.13
BAD16 0.12
BAD15 0.2
BAD14 0.12
BAD13 0.12
BAD12 0.12
BAD11 0.12
BAD06 0.14
BAD03 0.15
