# Supplementary material for: Genomic insights into the persistence of Nubian giraffe (Giraffa camelopardalis camelopardalis) in conflict-affected South Sudan
Source: Sci Rep. 2026 Sep 5;16:27825. doi: 10.1038/s41598-026-66559-1 (PMC13546283; doi:10.1038/s41598-026-66559-1)
Supplement: Supplementary file 3 [file 41598_2026_66559_MOESM4_ESM.pdf]

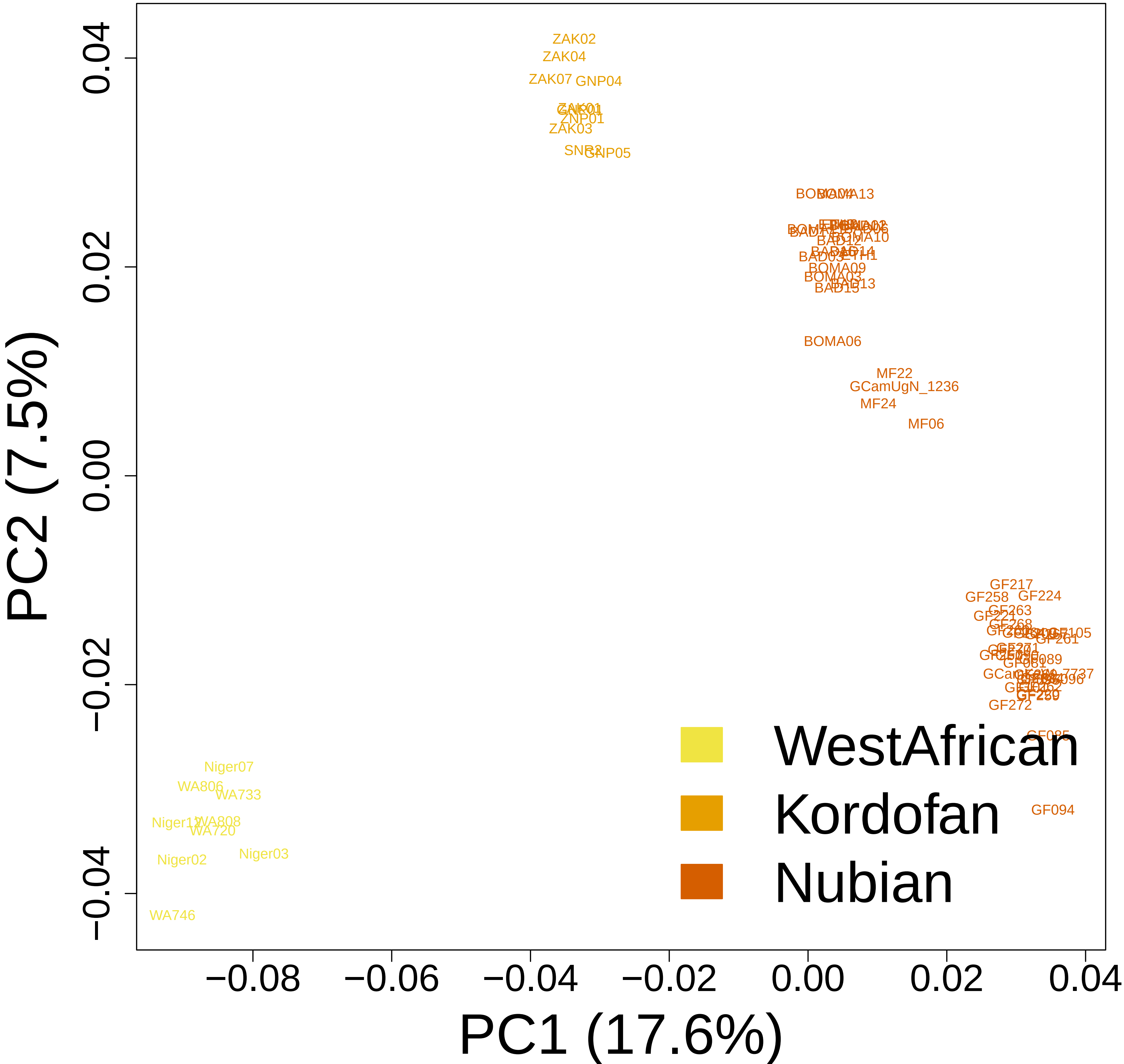

PC1 (17.6%)
PC2 (7.5%)
−0.08
−0.06
−0.04
−0.02
0.00
0.02
0.04
0.04
0.02
0.00
−0.02
−0.04
WestAfrican
Kordofan
Nubian
ZAK02
ZAK04
ZAK07
GNP04
ZAK03
GNP05
BOMA09
BOMA06
MF22
GCamUgN_1236
MF24
MF06
GF217
GF258
GF224
GF263
GF272
GF094
Niger07
WA806
WA733
WA720
Niger02
Niger03
WA746
