# Supplementary material for: Genomic insights into the persistence of Nubian giraffe (Giraffa camelopardalis camelopardalis) in conflict-affected South Sudan
Source: Sci Rep. 2026 Sep 5;16:27825. doi: 10.1038/s41598-026-66559-1 (PMC13546283; doi:10.1038/s41598-026-66559-1)
Supplement: Supplementary file 4 [file 41598_2026_66559_MOESM5_ESM.pdf]

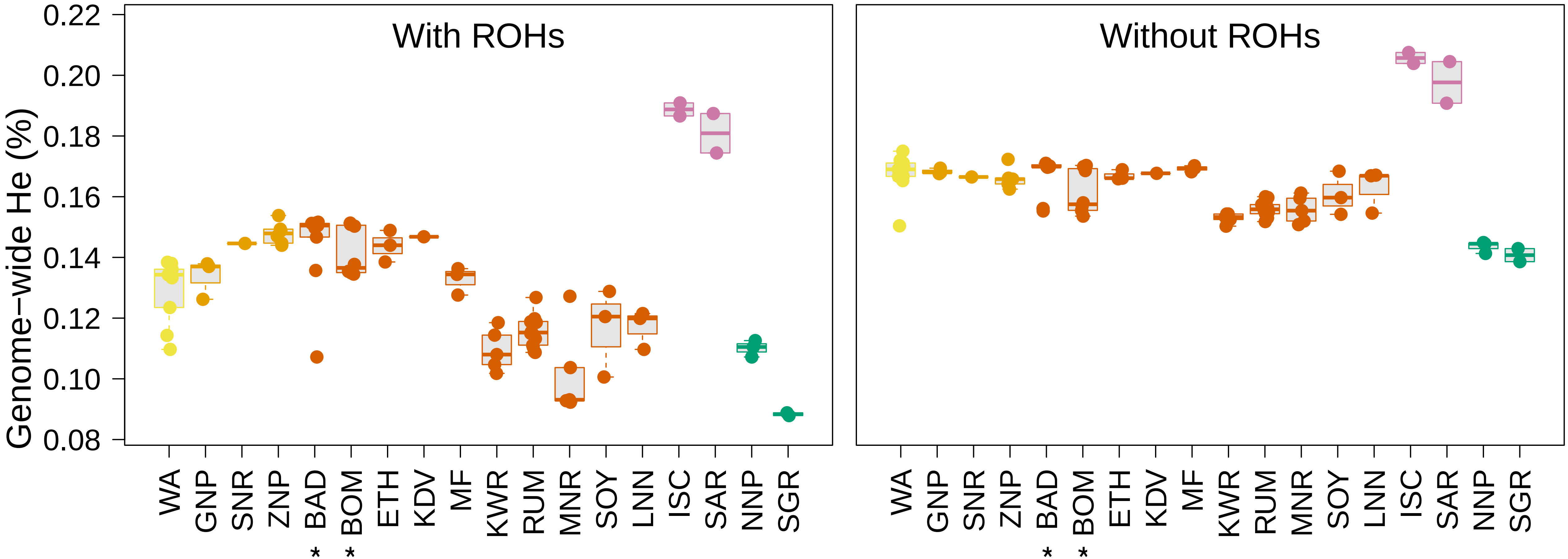
With ROHs
Without ROHs
Genome−wide He (%)
0.22
0.20
0.18
0.16
0.14
0.12
0.10
0.08
WA
GNP
SNR
ZNP
BAD
BOM
ETH
KDV
MF
KWR
RUM
MNR
SOY
LNN
ISC
SAR
NNP
SGR
*
*
