# Supplementary material for: Genomic insights into the persistence of Nubian giraffe (Giraffa camelopardalis camelopardalis) in conflict-affected South Sudan
Source: Sci Rep. 2026 Sep 5;16:27825. doi: 10.1038/s41598-026-66559-1 (PMC13546283; doi:10.1038/s41598-026-66559-1)
Supplement: Supplementary file 5 [file 41598_2026_66559_MOESM6_ESM.pdf]

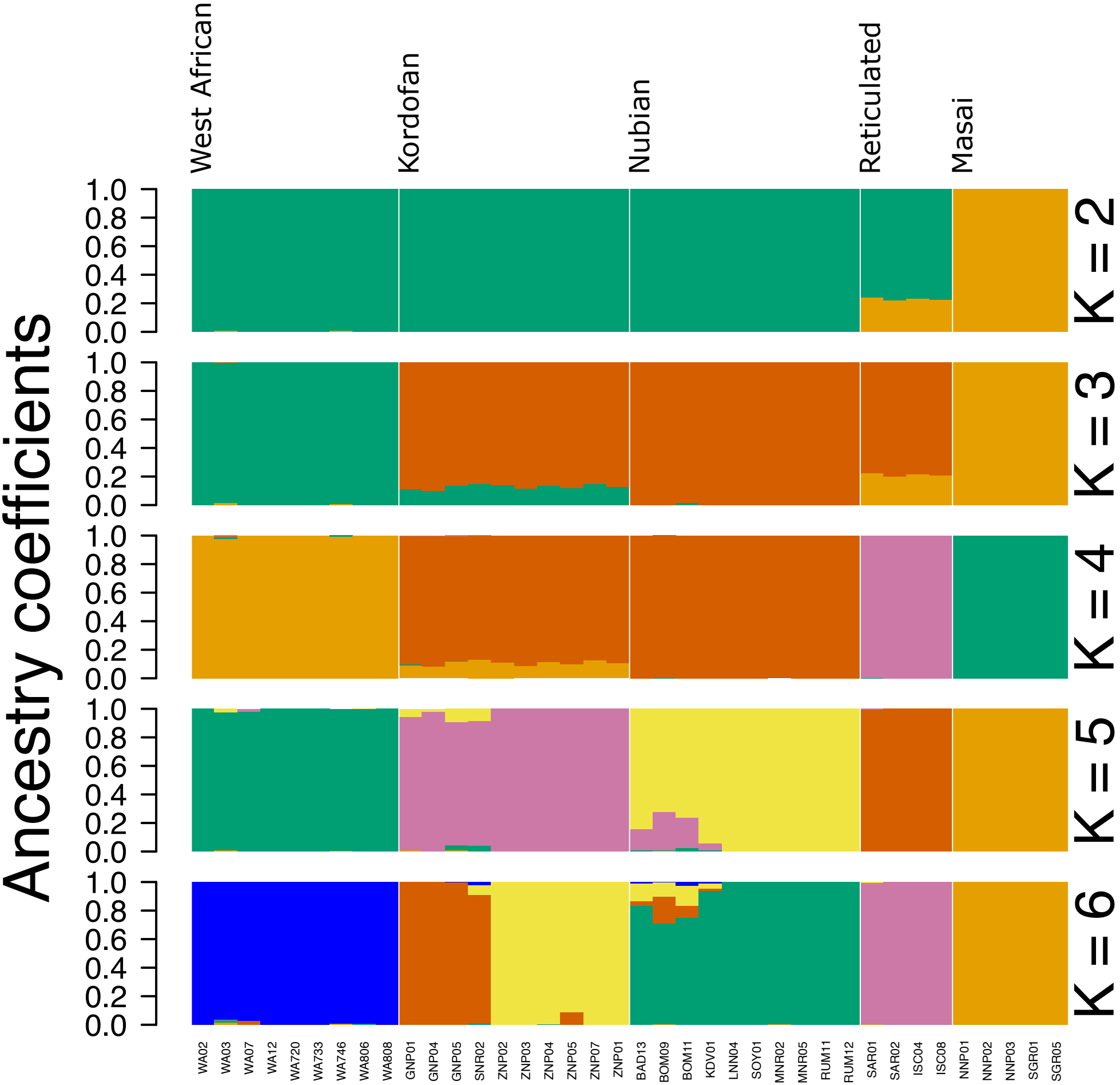

Ancestry coefficients
West African
Kordofan
Nubian
Reticulated
Masai
K = 2
K = 3
K = 4
K = 5
K = 6
1.0
0.8
0.6
0.4
0.2
0.0
WA02 WA03 WA07 WA12 WA720 WA733 WA746 WA806 WA808 GNP01 GNP04 GNP05 SNR02 ZNP02 ZNP03 ZNP04 ZNP05 ZNP07 ZNP01 BAD13 BOM09 BOM11 KDV01 LNN04 SOY01 MNR02 MNR05 RUM11 RUM12 SAR01 SAR02 ISC04 ISC08 NNP01 NNP02 NNP03 SGR01 SGR05
