# Supplementary material for: Genomic insights into the persistence of Nubian giraffe (Giraffa camelopardalis camelopardalis) in conflict-affected South Sudan
Source: Sci Rep. 2026 Sep 5;16:27825. doi: 10.1038/s41598-026-66559-1 (PMC13546283; doi:10.1038/s41598-026-66559-1)
Supplement: Supplementary file 6 [file 41598_2026_66559_MOESM7_ESM.pdf]

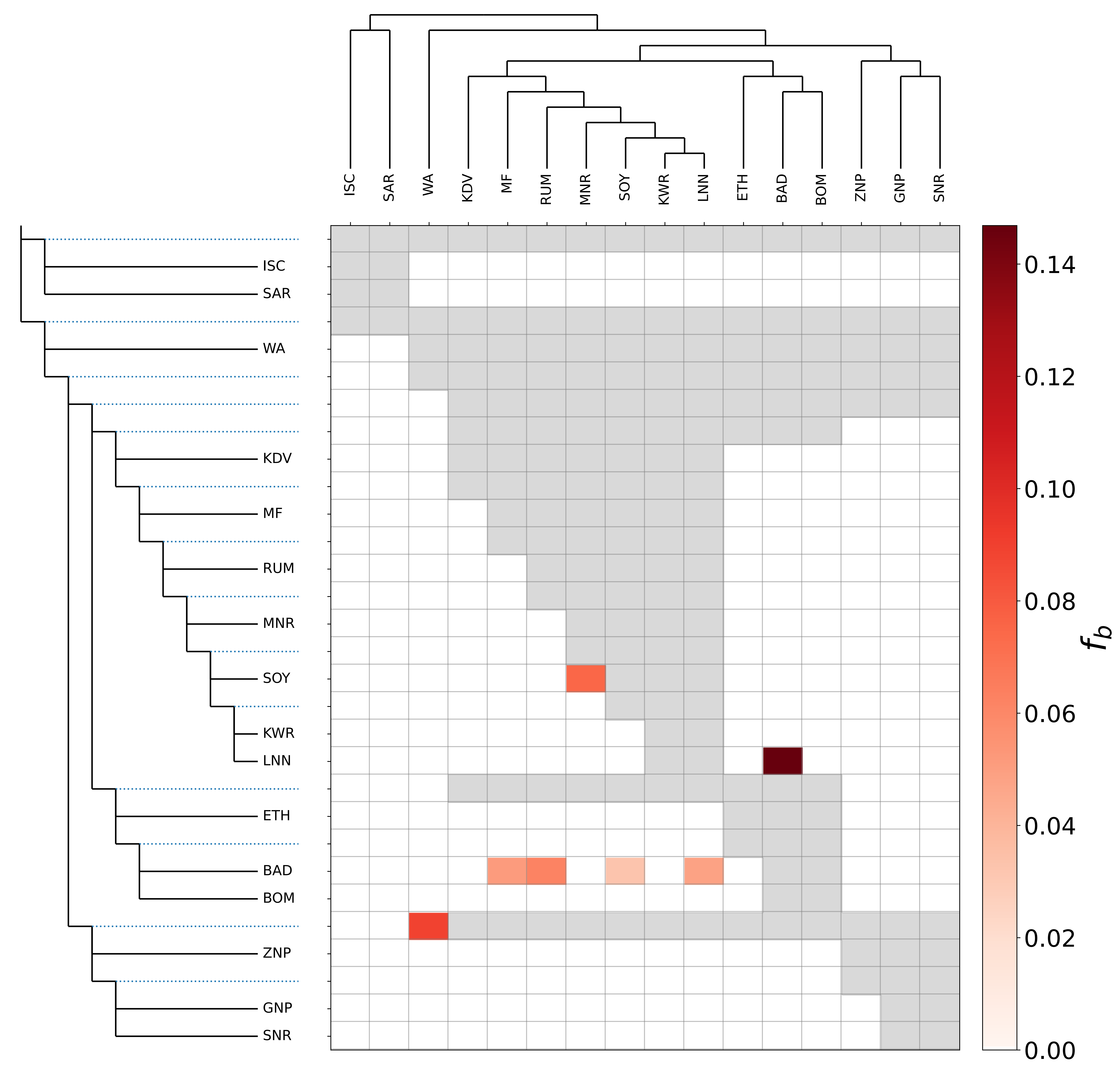

ISC
SAR
WA
KDV
MF
RUM
MNR
SOY
KWR
LNN
ETH
BAD
BOM
ZNP
GNP
SNR
f_b
0.00
0.02
0.04
0.06
0.08
0.10
0.12
0.14
