# Supplementary material for: Genomic insights into the persistence of Nubian giraffe (Giraffa camelopardalis camelopardalis) in conflict-affected South Sudan
Source: Sci Rep. 2026 Sep 5;16:27825. doi: 10.1038/s41598-026-66559-1 (PMC13546283; doi:10.1038/s41598-026-66559-1)
Supplement: Supplementary file 7 [file 41598_2026_66559_MOESM8_ESM.pdf]

Sample ID	Location	Taxonomy	Common name	SRA accession	Source	Total reads (10^6)	Dupped (%)	Depth (x)	Exclusion criterion
GNP01	Garamba NP, DR Congo	*Giraffa camelopardalis antiquorum*	Kordofan giraffe	SRR11905126	2	177,97	7,01	10,9	
GNP04	Garamba NP, DR Congo	*Giraffa camelopardalis antiquorum*	Kordofan giraffe	SRR11905125	2	204,30	6,81	12,6	
GNP05	Garamba NP, DR Congo	*Giraffa camelopardalis antiquorum*	Kordofan giraffe	SRR11905124	2	220,66	7,85	13,6	
SNR02	Shambe NP, South Sudan	*Giraffa camelopardalis antiquorum*	Kordofan giraffe	SRR11905123	2	186,93	7,08	11,5	
ZNP01	Zakouma NP, Chad	*Giraffa camelopardalis antiquorum*	Kordofan giraffe	ERR1248124	1	313,78	10,22	16,1	
ETH1	Gambella NP, Ethiopia	*Giraffa camelopardalis camelopardalis*	Nubian giraffe	SRR11905122	2	213,95	7,29	13,1	
ETH2	Gambella NP, Ethiopia	*Giraffa camelopardalis camelopardalis*	Nubian giraffe	SRR11905167	2	187,60	6,92	11,5	
ETH3	Gambella NP, Ethiopia	*Giraffa camelopardalis camelopardalis*	Nubian giraffe	SRR11905166	2	192,45	6,44	11,8	
KWR01	Kigio Wildlife CR, Kenya	*Giraffa camelopardalis camelopardalis*	Nubian giraffe	SRR18312683	5	95,44	3,57	5,9	
KWR02	Kigio Wildlife CR, Kenya	*Giraffa camelopardalis camelopardalis*	Nubian giraffe	SRR18312682	5	85,86	3,60	5,3	
KWR03	Kigio Wildlife CR, Kenya	*Giraffa camelopardalis camelopardalis*	Nubian giraffe	SRR18312681	5	7,91	0,86	0,5	Miss >0.5 and Relatedness
KWR04	Kigio Wildlife CR, Kenya	*Giraffa camelopardalis camelopardalis*	Nubian giraffe	SRR18312680	5	101,18	3,91	6,2	
KWR05	Kigio Wildlife CR, Kenya	*Giraffa camelopardalis camelopardalis*	Nubian giraffe	SRR18312679	5	103,18	3,87	6,3	
KWR06	Kigio Wildlife CR, Kenya	*Giraffa camelopardalis camelopardalis*	Nubian giraffe	SRR18312678	5	108,24	4,70	6,6	
LNN01	Lake Nakuru NP, Kenya	*Giraffa camelopardalis camelopardalis*	Nubian giraffe	SRR18312673	5	110,43	4,34	6,8	
LNN02	Lake Nakuru NP, Kenya	*Giraffa camelopardalis camelopardalis*	Nubian giraffe	SRR18312672	5	205,85	7,42	12,6	
LNN03	Lake Nakuru NP, Kenya	*Giraffa camelopardalis camelopardalis*	Nubian giraffe	SRR18312671	5	26,33	3,87	1,6	Miss >0.5
MF06	Murchison Falls NP, Uganda	*Giraffa camelopardalis camelopardalis*	Nubian giraffe	SRR11905165	2	180,67	6,05	11,1	
MF22	Murchison Falls NP, Uganda	*Giraffa camelopardalis camelopardalis*	Nubian giraffe	SRR11905164	2	276,45	8,79	17,0	
MF24	Murchison Falls NP, Uganda	*Giraffa camelopardalis camelopardalis*	Nubian giraffe	SRR11905163	2	288,85	9,17	17,7	
MNR01	Mwea NR, Kenya	*Giraffa camelopardalis camelopardalis*	Nubian giraffe	SRR18312644	5	106,02	3,82	6,5	
MNR02	Mwea NR, Kenya	*Giraffa camelopardalis camelopardalis*	Nubian giraffe	SRR18312639	5	106,33	3,93	6,5	
MNR03	Mwea NR, Kenya	*Giraffa camelopardalis camelopardalis*	Nubian giraffe	SRR18312637	5	125,54	4,56	7,7	
MNR04	Mwea NR, Kenya	*Giraffa camelopardalis camelopardalis*	Nubian giraffe	SRR18312636	5	148,98	5,27	9,2	
MNR05	Mwea NR, Kenya	*Giraffa camelopardalis camelopardalis*	Nubian giraffe	SRR18312634	5	96,33	4,85	5,9	
RUM01	Ruma NP, Kenya	*Giraffa camelopardalis camelopardalis*	Nubian giraffe	SRR18312610	5	122,85	4,72	7,5	
RUM02	Ruma NP, Kenya	*Giraffa camelopardalis camelopardalis*	Nubian giraffe	SRR18312609	5	122,93	4,56	7,6	
RUM03	Ruma NP, Kenya	*Giraffa camelopardalis camelopardalis*	Nubian giraffe	SRR18312607	5	96,38	4,23	5,9	
RUM04	Ruma NP, Kenya	*Giraffa camelopardalis camelopardalis*	Nubian giraffe	SRR18312606	5	114,89	4,07	7,1	
RUM05	Ruma NP, Kenya	*Giraffa camelopardalis camelopardalis*	Nubian giraffe	SRR18312605	5	97,01	3,49	6,0	
RUM06	Ruma NP, Kenya	*Giraffa camelopardalis camelopardalis*	Nubian giraffe	SRR18312604	5	128,11	4,69	7,9	
RUM07	Ruma NP, Kenya	*Giraffa camelopardalis camelopardalis*	Nubian giraffe	SRR18312603	5	111,96	4,27	0,9	
RUM08	Ruma NP, Kenya	*Giraffa camelopardalis camelopardalis*	Nubian giraffe	SRR18312602	5	109,99	4,39	6,8	
RUM09	Ruma NP, Kenya	*Giraffa camelopardalis camelopardalis*	Nubian giraffe	SRR18312601	5	113,22	4,42	7,0	
RUM10	Ruma NP, Kenya	*Giraffa camelopardalis camelopardalis*	Nubian giraffe	SRR18312600	5	106,26	3,98	6,5	
RUM11	Ruma NP, Kenya	*Giraffa camelopardalis camelopardalis*	Nubian giraffe	SRR18312599	5	119,83	4,28	7,4	
RUM12	Ruma NP, Kenya	*Giraffa camelopardalis camelopardalis*	Nubian giraffe	SRR18312598	5	113,76	4,34	7,0	
RUM13	Ruma NP, Kenya	*Giraffa camelopardalis camelopardalis*	Nubian giraffe	SRR18312708	5	117,76	4,40	7,2	
RUM14	Ruma NP, Kenya	*Giraffa camelopardalis camelopardalis*	Nubian giraffe	SRR18312707	5	104,17	3,89	6,4	
SOY01	Soysambu CR, Kenya	*Giraffa camelopardalis camelopardalis*	Nubian giraffe	SRR18312684	5	135,53	4,94	8,3	
SOY02	Soysambu CR, Kenya	*Giraffa camelopardalis camelopardalis*	Nubian giraffe	SRR18312677	5	279,45	9,21	17,2	
SOY03	Soysambu CR, Kenya	*Giraffa camelopardalis camelopardalis*	Nubian giraffe	SRR18312676	5	99,72	3,75	6,1	
SOY04	Soysambu CR, Kenya	*Giraffa camelopardalis camelopardalis*	Nubian giraffe	SRR18312675	5	62,99	3,11	3,9	Miss >0.5
WA720	Koure Plateau, Niger	*Giraffa camelopardalis peralta*	West African giraffe	SRR11905169	2	202,37	6,76	12,4	
WA733	Koure Plateau, Niger	*Giraffa camelopardalis peralta*	West African giraffe	SRR11905168	2	165,13	5,52	10,1	
WA746	Koure Plateau, Niger	*Giraffa camelopardalis peralta*	West African giraffe	SRR11905157	2	147,46	5,05	9,1	
WA806	Koure Plateau, Niger	*Giraffa camelopardalis peralta*	West African giraffe	SRR11905146	2	196,57	6,58	12,1	
WA808	Koure Plateau, Niger	*Giraffa camelopardalis peralta*	West African giraffe	SRR11905135	2	187,81	6,22	11,5	
ISC01	Ishaqbini CR, Kenya	*Giraffa reticulata*	Reticulated giraffe	SRR11905154	2	72,70	4,86	4,4	Miss >0.5
ISC04	Ishaqbini CR, Kenya	*Giraffa reticulata*	Reticulated giraffe	SRR11905153	2	174,43	6,24	10,7	
ISC08	Ishaqbini CR, Kenya	*Giraffa reticulata*	Reticulated giraffe	SRR11905152	2	228,58	7,60	14,0	
SAR01	Samburu NR, Kenya	*Giraffa reticulata*	Reticulated giraffe	SRR18312638	5	108,41	4,01	6,7	
SAR02	Samburu NR, Kenya	*Giraffa reticulata*	Reticulated giraffe	SRR18312635	5	141,84	5,11	8,7	
SGR01	Selous GR, Tanzania	*Giraffa tippelskirchi tippelskirchi*	Masai giraffe	SRR11905144	2	185,52	6,08	11,4	
SGR05	Selous GR, Tanzania	*Giraffa tippelskirchi tippelskirchi*	Masai giraffe	SRR11905143	2	278,39	8,80	17,1	
NNP01	Nairobi NP, Kenya	*Giraffa tippelskirchi tippelskirchi*	Masai giraffe	SRR18312711	5	101,75	4,52	6,3	
NNP02	Nairobi NP, Kenya	*Giraffa tippelskirchi tippelskirchi*	Masai giraffe	SRR18312710	5	118,38	4,42	7,3	
NNP03	Nairobi NP, Kenya	*Giraffa tippelskirchi tippelskirchi*	Masai giraffe	SRR18312685	5	104,38	3,78	6,4	
LNN04	Lake Nakuru NP, Kenya	*Giraffa camelopardalis camelopardalis*	Nubian giraffe	SAMEA114401683*	4	283,19	9,51	17,4	
KDV01	Kidepo Valley NP, Uganda	Giraffa camelopardalis camelopardalis	Nubian giraffe	SAMEA114401671*	4	239,46	11,85	14,5	
WA02	Kouré, Niger	Giraffa camelopardalis peralta	West African giraffe	SRR16477187	3	151,61	5,05	9,3	
WA03	Kouré, Niger	Giraffa camelopardalis peralta	West African giraffe	SRR16477186	3	76,73	2,75	4,7	
WA06	Kouré, Niger	Giraffa camelopardalis peralta	West African giraffe	SRR16477185	3	156,69	5,32	9,6	Relatedness
WA07	Kouré, Niger	Giraffa camelopardalis peralta	West African giraffe	SRR16477184	3	141,50	4,81	8,7	
WA12	Kouré, Niger	Giraffa camelopardalis peralta	West African giraffe	SRR16477183	3	138,54	4,69	8,5	
ZNP02	Zakouma NP, Chad	Giraffa camelopardalis antiquorum	Kordofan giraffe	SRR16477182	3	155,04	5,16	9,5	
ZNP03	Zakouma NP, Chad	Giraffa camelopardalis antiquorum	Kordofan giraffe	SRR16477181	3	134,31	4,54	8,3	
ZNP04	Zakouma NP, Chad	Giraffa camelopardalis antiquorum	Kordofan giraffe	SRR16477180	3	153,67	5,1	9,4	
ZNP05	Zakouma NP, Chad	Giraffa camelopardalis antiquorum	Kordofan giraffe	SRR16477179	3	155,83	5,29	9,6	
ZNP06	Zakouma NP, Chad	Giraffa camelopardalis antiquorum	Kordofan giraffe	SRR16477178	3	155,40	5,25	9,5	
BAD01	Badingilo NP, South Sudan	*Giraffa camelopardalis camelopardalis*	Nubian giraffe	this study	this study	102,61	16,12	6,0	Miss >0.5
BAD02	Badingilo NP, South Sudan	*Giraffa camelopardalis camelopardalis*	Nubian giraffe	this study	this study	83,44	14,44	4,8	Miss >0.5
BAD03	Badingilo NP, South Sudan	*Giraffa camelopardalis camelopardalis*	Nubian giraffe	this study	this study	116,57	7,62	7,1	
BAD04	Badingilo NP, South Sudan	*Giraffa camelopardalis camelopardalis*	Nubian giraffe	this study	this study	53,79	17,76	3,1	Miss >0.5
BAD05	Badingilo NP, South Sudan	*Giraffa camelopardalis camelopardalis*	Nubian giraffe	this study	this study	74,16	16,71	4,2	Miss >0.5
BAD06	Badingilo NP, South Sudan	*Giraffa camelopardalis camelopardalis*	Nubian giraffe	this study	this study	120,98	6,59	7,4	
BAD07	Badingilo NP, South Sudan	*Giraffa camelopardalis camelopardalis*	Nubian giraffe	this study	this study	77,35	16,55	4,4	Miss >0.5
BAD08	Badingilo NP, South Sudan	*Giraffa camelopardalis camelopardalis*	Nubian giraffe	this study	this study	82,34	17,50	4,7	Miss >0.5
BAD09	Badingilo NP, South Sudan	*Giraffa camelopardalis camelopardalis*	Nubian giraffe	this study	this study	111,00	19,62	6,4	Miss >0.5
BOM01	Boma NP, South Sudan	*Giraffa camelopardalis camelopardalis*	Nubian giraffe	this study	this study	97,93	17,53	5,7	Miss >0.5
BOM02	Boma NP, South Sudan	*Giraffa camelopardalis camelopardalis*	Nubian giraffe	this study	this study	128,66	7,09	7,9	
BOM03	Boma NP, South Sudan	*Giraffa camelopardalis camelopardalis*	Nubian giraffe	this study	this study	112,21	6,84	6,8	
BOM04	Boma NP, South Sudan	*Giraffa camelopardalis camelopardalis*	Nubian giraffe	this study	this study	109,10	5,80	6,7	
BOM05	Boma NP, South Sudan	*Giraffa camelopardalis camelopardalis*	Nubian giraffe	this study	this study	116,35	10,16	7,1	Miss >0.5
BAD10	Badingilo NP, South Sudan	*Giraffa camelopardalis camelopardalis*	Nubian giraffe	this study	this study	71,10	22,47	4,1	Miss >0.5
BOM06	Boma NP, South Sudan	*Giraffa camelopardalis camelopardalis*	Nubian giraffe	this study	this study	144,35	11,99	8,8	
BOM07	Boma NP, South Sudan	*Giraffa camelopardalis camelopardalis*	Nubian giraffe	this study	this study	73,18	18,52	4,2	Miss >0.5
BOM08	Boma NP, South Sudan	*Giraffa camelopardalis camelopardalis*	Nubian giraffe	this study	this study	84,97	20,98	4,8	Miss >0.5
BOM09	Boma NP, South Sudan	*Giraffa camelopardalis camelopardalis*	Nubian giraffe	this study	this study	110,75	6,17	6,8	
BOM10	Boma NP, South Sudan	*Giraffa camelopardalis camelopardalis*	Nubian giraffe	this study	this study	329,78	2,24	20,2	
BOM11	Boma NP, South Sudan	*Giraffa camelopardalis camelopardalis*	Nubian giraffe	this study	this study	404,39	2,66	24,8	
BAD11	Badingilo NP, South Sudan	*Giraffa camelopardalis camelopardalis*	Nubian giraffe	this study	this study	350,97	1,97	21,5	
BAD12	Badingilo NP, South Sudan	*Giraffa camelopardalis camelopardalis*	Nubian giraffe	this study	this study	371,65	0,51	22,8	
BAD13	Badingilo NP, South Sudan	*Giraffa camelopardalis camelopardalis*	Nubian giraffe	this study	this study	373,16	0,57	22,8	
BAD14	Badingilo NP, South Sudan	*Giraffa camelopardalis camelopardalis*	Nubian giraffe	this study	this study	355,03	0,63	21,8	
BAD15	Badingilo NP, South Sudan	*Giraffa camelopardalis camelopardalis*	Nubian giraffe	this study	this study	387,03	0,5	23,7	
BAD16	Badingilo NP, South Sudan	*Giraffa camelopardalis camelopardalis*	Nubian giraffe	this study	this study	416,82	0,58	25,5	
BAD17	Badingilo NP, South Sudan	*Giraffa camelopardalis camelopardalis*	Nubian giraffe	this study	this study	337,47	0,43	20,7	
BOM12	Boma NP, South Sudan	*Giraffa camelopardalis camelopardalis*	Nubian giraffe	this study	this study	395,38	0,55	24,2	Relatedness
BOM13	Boma NP, South Sudan	*Giraffa camelopardalis camelopardalis*	Nubian giraffe	this study	this study	360,66	0,54	22,1	

* Sample ID from European Nucleotide Archive (ENA)

References

1. Fennessy J, Bidon T, Reuss F, Kumar V, Elkan P, Nilsson MA, et al. Multi-locus analyses reveal four giraffe species instead of one. Curr Biol. 2016;26(18):2543-2549.
2. Coimbra RTF, Winter S, Kumar V, Koepfli KP, Gooley RM, Dobrynin P, et al. Whole-genome analysis of giraffe supports four distinct species. Curr Biol. 2021;31(13):2929-2938.e5.
3. Coimbra, R. T. F., Winter, S., Mitchell, B., Fennessy, J., & Janke, A. (2022). Conservation Genomics of Two Threatened Subspecies of Northern Giraffe: The West African and the Kordofan Giraffe. Genes, 13(2), 221. https://doi.org/10.3390/genes13020221
4. Bertola, L. D., Quinn, L., Hanghøj, K., Garcia-Erill, G., Rasmussen, M. S., Balboa, R. F et al. Giraffe lineages are shaped by major ancient admixture events. Curr Biol. 2024;34(7), 1576-1586.
5. Coimbra, R.T.F., Winter, S., Muneza, A. *et al.* Genomic analysis reveals limited hybridization among three giraffe species in Kenya. *BMC Biol* **21**, 215 (2023). https://doi.org/10.1186/s12915-023-01722-y
