# Supplementary material for: Genomic insights into the persistence of Nubian giraffe (Giraffa camelopardalis camelopardalis) in conflict-affected South Sudan
Source: Sci Rep. 2026 Sep 5;16:27825. doi: 10.1038/s41598-026-66559-1 (PMC13546283; doi:10.1038/s41598-026-66559-1)
Supplement: Supplementary file 8 [file 41598_2026_66559_MOESM1_ESM.docx]

SUPPLEMENTARY MATERIALS

**Genomic insights into the persistence of Nubian giraffe (Giraffa camelopardalis camelopardalis) in conflict-affected South Sudan**

Sam van Giessen^1^, David Prochotta^2^, Menno Jeroen de Jong^2^, Richard Harvey^3^, Megan Claase^4^, Julian Fennessy^5,6^, Axel Janke ^2, 7*^

***Table S1: Sample map (Fig. 1A) legend, sample IDs, and origin.***

| **Location Label** | **Location** | | **Country** | **(Sub)species** | **N** | **Retained N** |
| --- | --- | --- | --- | --- | --- | --- |
| WA | Kouré Plateau | Niger | | *G.c. peralta* | 10 | 9 |
| ZNP | Zakouma NP | Chad | | *G.c. antiquorum* | 6 | 6 |
| SNR | Shambe NP | South Sudan | | *G.c. antiquorum* | 1 | 1 |
| GNP | Garamba NP | Democratic Republic Congo | | *G.c. antiquorum* | 3 | 3 |
| ETH | Gambella NP | Ethiopia | | *G.c. camelopardalis* | 3 | 3 |
| BOM | Boma NP* | South Sudan | | *G.c. camelopardalis* | 13 | 8 |
| BAD | Badingilo NP* | South Sudan | | *G.c. camelopardalis* | 17 | 9 |
| KDV | Kidepo Valley NP | Uganda | | *G.c. camelopardalis* | 1 | 1 |
| MF | Murchison Falls NP | Uganda | | *G.c. camelopardalis* | 3 | 3 |
| RUM | Ruma NP | Kenya | | *G.c. camelopardalis* | 14 | 14 |
| SOY | Soysambu CR | Kenya | | *G.c. camelopardalis* | 4 | 3 |
| LNN | Lake Nakuru NP | Kenya | | *G.c. camelopardalis* | 4 | 3 |
| KWR | Kigio Wildlife CR | Kenya | | *G.c. camelopardalis* | 6 | 5 |
| MNR | Mwea NR | Kenya | | *G.c. camelopardalis* | 5 | 5 |
| SAR | Samburu NR | Kenya | | *G. reticulata* | 2 | 2 |
| ISC | Ishaqbini Hirola CR | Kenya | | *G. reticulata* | 3 | 2 |
| NNP | Nairobi NP | Kenya | | *G. tippelskirchi tippelskirchi* | 3 | 3 |
| SGR | Selous Game Reserve | Tanzania | | *G. tippelskirchi tippelskirchi* | 2 | 2 |
|  |  |  | | **Total** | 100 | 82 |

***Table S2: Sample information and genome coverage summary table****. In the Exclusion criterion column the* *reason for exclusion from all analyses is noted (continues next page).*

**Figure S1: Depth distribution plot.** The plot shows the distribution of the read depth of sites in the VCF file and the effect of the corresponding filtering settings. The left red line shows the minimum site depth of 100 and the right line shows the maximum site depth of 1800.

 ***Figure S2: Mean mapped read depth per site in the VCF of individuals before SNP filtering.*** *The samples are grouped per sampled location.*

***Figure S3: Heterozygosity on the second chromosome.*** *Plot shows the heterozygosity over the second chromosome of all individuals, areas identified as ROHs by Darwindow are marked grey. This plot is used to verify correct parameter setting of the ROH identification.*

***Figure S4: PCoA plot of northern giraffe.*** *The analysis, excluding the outgroup for better resolution, confirms the dendrogram analysis and identifies the same clusters according to subspecies and geographic origin. (Labels, see Table S1)*

***Figure S5: Comparison plot of the genome-wide heterozygosity levels (%) per population.*** *In the left panel segments of the genome identified by Darwindow as ROHs are included and in the right-side panel these segments are excluded.*

***Figure S6: Cross entropy plot of LEA of whole dataset.*** *This plot is used to find an optimal value of ancestral populations which are enforced in the admixture analysis (Fig 3A). The plot shows an optimal (lowest minimal cross entropy) for K=5.*

***Figure S7: Admixture plot for K = 2 - 6 of the thinned dataset where Nubian giraffe is randomly down sampled to 10 individuals.*** *Each vertical bar represents one individual from the five (Sub)species; Kordofan, Masai, Nubian, reticulated, and* *West African giraffe. The colours represent the division of the individuals into a specified number of genetic clusters (K). Individuals with mixed coloured bars indicate admixture, or artifacts of admixture when too few genetic clusters are imposed on the data. At the optimal K = 4 (see Fig S8) the Kordofan, Masai, reticulated, and West African giraffe show little very admixture. The Nubian and Kordofan giraffe show admixed ancestry. At K = 6 the Nubian giraffe from South Sudan and Uganda show admixed ancestry with the Kordofan giraffe.*

***Figure S8: Cross entropy plot of LEA of thinned dataset where Nubian giraffe is randomly down sampled to 10 individuals.*** *This plot is used to find an optimal value of ancestral populations which are enforced in the admixture analysis. The plot shows an optimal (lowest minimal cross entropy) for K=4.*

***Figure S9: Heatmap of estimated gene flow between different combinations of branches of the phylogeny of each population in dataset.*** *The darker coloured cells indicate higher levels of gene flow (f_b_). The only gene flow between subspecies seen in this analysis is between the ancestral Kordofan group (ZNP, GNP, SNR) and those from Niger (WA).*
